# Potential Antitumor Effect of Harmine in the Treatment of Thyroid Cancer

**DOI:** 10.1155/2017/9402615

**Published:** 2017-02-08

**Authors:** Shu Ruan, Feng Jia, Jianbo Li

**Affiliations:** ^1^Department of Endocrinology, The First Affiliated Hospital of Nanjing Medical University, Nanjing, China; ^2^Department of Endocrinology, Yancheng Third Hospital, The Affiliated Hospital of Southeast University Medical College, Yancheng, China; ^3^Department of Neurosurgery, Yancheng City No. 1 People's Hospital, The Fourth Affiliated Hospital of Nantong Medical College, Yancheng, China

## Abstract

Thyroid cancer is one of the most common types of cancer in endocrine system. In latest studies, harmine has been proved to inhibit the growth of several cancers in vitro and in vivo. In the current study, we evaluated the in vitro and in vivo anticancer efficiency of harmine against thyroid cancer cell line TPC-1. The in vitro cytotoxicity of harmine evaluated by XTT assay indicated that harmine suppressed the proliferation of TPC-1 cells in a dose- and time-dependent manner. Moreover, harmine dose-dependently induced apoptosis of TPC-1 cells through regulating the ratio of Bcl-2/Bax. The colony forming ability of TPC-1 cells was also time-dependently inhibited by harmine. The inhibitory effects of harmine on migration and invasion of TPC-1 cells were studied by wound scratching and Transwell assays. In vivo evaluation showed that harmine effectively inhibited the growth of thyroid cancer in a dose-dependent manner in nude mice. Therefore, harmine might be a promising herbal medicine in the therapy of thyroid cancer and further efforts are needed to explore this therapeutic strategy.

## 1. Introduction

Thyroid cancer is one of the most common kinds of cancer in endocrine system which includes four major types with papillary thyroid cancer (PTC) being the most common one [1]. Though most of PTCs respond well to surgery and chemoradiotherapy, part of them quickly develop resistance to chemotherapy, which generate an urgent demand of novel strategy to improve the efficacy of chemotherapeutics [2].

Recently a growing body of evidences demonstrated the promising antitumor efficiency of herbal medicine [3–5]. Several herbal medicines, such as harmine, resveratrol, and berbamine, have shown substantial tumor inhibitory effect against a series of cancers [6–8]. Among all these herbal medicines, harmine, originally isolated from the seeds of* Peganum harmala*, is a tricyclic compound belonging to the *β*-carboline alkaloids [9]. It is reported that harmine possesses various pharmacological activities [10]. In latest studies, harmine has been proved to inhibit the growth of several cancers in vitro and in vivo [11, 12]. For example, harmine induces the apoptosis of cancer cells by leading to DNA break as demonstrated in DNA fragmentation and tunnel study in melanoma cells [13]. Possible mechanisms include the block of homologous recombination, one of the major Double Strand Break (DSB) repair pathways and the inhibition of telomerase activity [9, 12]. However, no related reports were found on the antitumor effect of harmine in thyroid cancer.

In the current study, we evaluated the in vitro and in vivo anticancer efficiency of harmine against thyroid cancer. The cell inhibition effect of harmine was measured by XTT assay. Apoptosis of thyroid cancer cell line TPC-1 was measured by DAPI staining. Related apoptotic protein expression was evaluated by western blotting. Influences of harmine on the migration and invasion of TPC-1 cells were also examined by wound scratching and Transwell assays. The in vivo antitumor effect was examined in a xenograft model of thyroid cancer.

## 2. Materials and Methods

### 2.1. Materials

Harmine was purchased from Sigma Co., Ltd. (St. Louis, USA). Harmine was dissolved in DMSO to make a stock solution at a concentration of 10 mg/mL. Working solution was prepared by diluting the stock solution into medium with the final concentration of DMSO below 0.1%. Human papillary thyroid cancer cell line TPC-1 was purchased from the Institute of Biochemistry and Cell Biology, Chinese Academy of Sciences (Shanghai, China). TPC-1 cells were cultured in RPMI 1640 (w L-glutamate) completed by fetal bovine serum (10%) and penicillin/streptomycin (1%) at 37°C in a humidified atmosphere containing 5% CO_2_.

Male and female nude mice (nu/nu; 6–8 weeks old and weighing 18–22 g) were purchased from the Animal Center of Nanjing Medical University (Nanjing, China). The animal protocol was reviewed and approved by the Institutional Animal Care and Use Committee of Nanjing Medical University.

### 2.2. In Vitro Cytotoxicity

TPC-1 cells were seeded in 96-well plates with a density of 10000 cells per well and allowed to grow 24 h before treatment. Cells were then incubated with escalated doses of harmine (2, 4, 8, 16, 32, and 64 *μ*g/mL) for 24, 36, and 48 h. The medium was replaced with 50 *μ*L XTT working solution and incubated for another 18 h. The optical density of each well was measured on a microplate reader at 560 nm (Bio-Rad, Hercules, USA).

### 2.3. DAPI Staining

Cells were seeded on cover slips in a 24-well plate and allowed to grow 24 h. Cells were then exposed to three kinds of concentration of harmine (4, 8, and 16 *μ*g/mL) for 36 h. After being washed by PBS, cells were fixed by a mixture of cold methanol and acetone (1 : 1) for 5 min. 4 *μ*g/mL 4′,6-diamidine-2′-phenylindole dihydrochloride (DAPI) was added to the well and allowed to incubate for 10 min at room temperature. Cells were imaged under a fluorescent microscope.

### 2.4. Western Blot

TPC-1 cells were incubated with harmine (4, 8, and 16 *μ*g/mL) for 6 hrs. Cell total proteins were extracted and quantified according to the instruction of the kit. 50 *μ*g of proteins was loaded into each well during the electrophoresis. Protein expression was measured by western blot as reported previously. Primary antibodies (anti-Bax, anti-Bcl-2, and anti-*β*-actin) were purchased from Sigma Co., Ltd. (St. Louis, MO, USA).

### 2.5. Clonogenic Assay

The first clonogenic assay was performed to study the inhibition of harmine on the colony forming ability of TPC-1 cells. Cells were treated with harmine (4, 8, and 16 *μ*g/mL) for 24, 36, and 48 h. Another clonogenic assay was performed to evaluate the radiosensitizing effect of harmine. TPC-1 cells were treated with 2 *μ*g/mL harmine and escalated doses of radiation singly or in combination. After each treatment, cells were trypsinized, collected, and set to the calculated concentration. After 10–14 days of incubation in 6-well plates, the 6-well plates were dyed by 0.5% crystal violet and counted as reported previously [14].

### 2.6. DNA Fragmentation Assay

TPC-1 cells were seeded in a 24-well plate and allowed to grow for 24 h. Cells were then exposed to three kinds of concentration of harmine (4, 8, and 16 *μ*g/mL) for 36 h. Total DNA was extracted and run by gel electrophoresis as previously reported [15].

### 2.7. Wound Scratching Assay and Transwell Assay

Cells were seeded in 6-well plate and allowed to grow for 24 h to form a monolayer. Then a tip was utilized to scratch in the middle to generate a clear space (wound). Immediately it is captured by the camera and was designated as 0 h. Then the medium was replaced with serum-free medium containing different concentrations of harmine (4, 8, and 16 *μ*g/mL). After 36 h, another image was captured to measure the width of the wound. The wound healing rate was calculated by the following formula: the average width of wound at 0 h − the average width of wound at 36 h/the average width of wound at 0 h.

Transwell assays were performed in 24-well Transwell chambers (Corning, Acton, MA, USA) as reported previously. Briefly, TPC-1 cells were seeded in the upper chamber in serum-free medium, while the bottom chamber was filled with complete medium as an attractant. 36 h later, the successfully invaded cells on the bottom chamber were stained by crystal violet, counted, and plotted.

### 2.8. In Vivo Antitumor Efficacy

Briefly, 1.5 million TPC-1 cells were injected subcutaneously in the left axillary space of the mice. 10 days later the mice with the tumor nodule reaching a volume of 70–80 mm^3^ were randomly divided into four groups with 6 mice per group. Escalated doses of harmine were administered by tail vein injection.

The mice were then treated with escalated doses of harmine with saline as the control. Tumor volume was measured by a caliper every other day during the whole experiment. The tumor volume (TV) was calculated with the following formula: TV = (*W*^2^*∗L*)/2, where *W* is the width of tumor nodule and *L* is the length of tumor nodule.

### 2.9. Statistical Analysis

Results were presented as mean ± SD. Statistical analysis was made by Student's *t*-test or ANOVA. The *p* value < 0.05 was considered as significant.

## 3. Results

### 3.1. Cytotoxicity and Apoptotic Induction of Harmine on TPC-1 Cells

**Figure 1:**
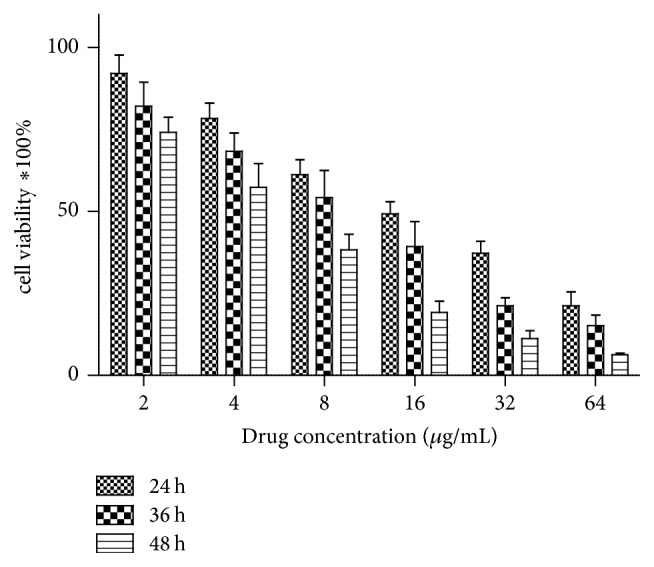
In vitro cytotoxicity of harmine on TPC-1 cells for the incubation of 24, 36, and 48 hrs.

As shown in Figure 1, escalated doses of harmine effectively led to the decrease of cell viability at different incubation times (Figure 1). The IC50 values of harmine against TPC-1 cells at 24, 36, and 48 h were 16.57 ± 1.4, 9.48 ± 1.1, and 5.51 ± 0.7 *μ*g/mL, respectively. It is noted that the IC50 values significantly lowered as the incubation time extended. Therefore, harmine inhibited the proliferation of TPC-1 cells in a time- and dose-dependent manner.

**Figure 2:**
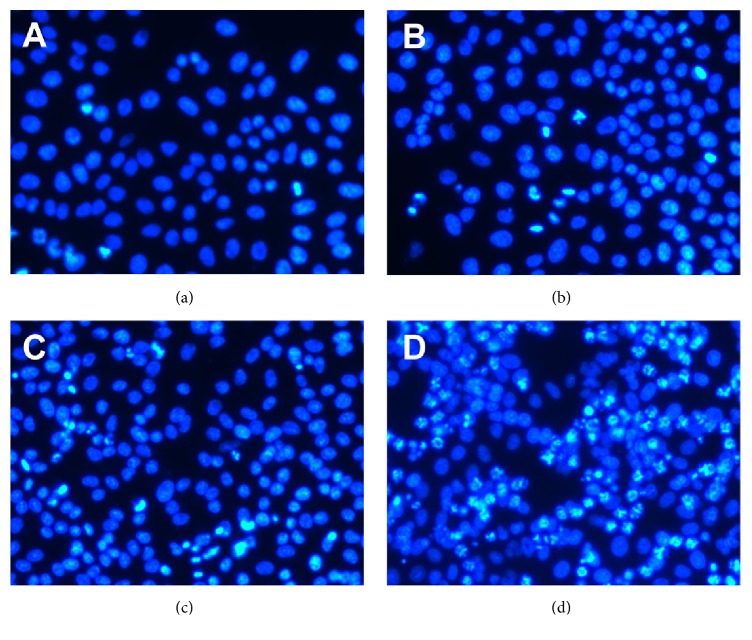
Apoptosis of TPC-1 cells detected by DAPI staining. (a) The nontreated cells. (b) Cells were treated with 4 *μ*g/mL harmine for 36 h. (c) Cells were treated with 8 *μ*g/mL harmine for 36 h. (d) Cells were treated with 16 *μ*g/mL harmine for 36 h.

**Figure 3:**
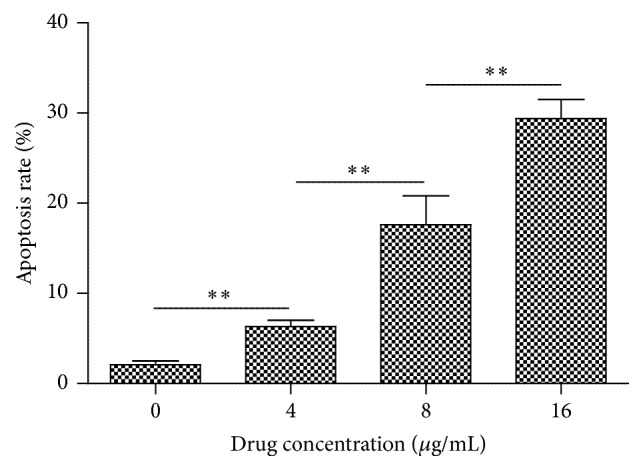
Quantitative analysis of apoptotic rate of cells exposed to different agents. Values are represented as mean ± SD (*n* = 3). *∗∗* means *p* < 0.01.

**Figure 4:**
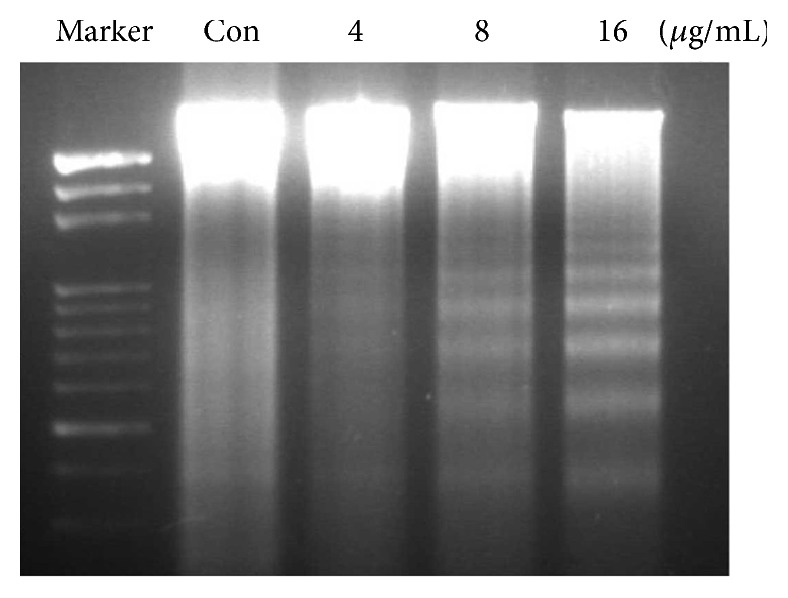
DNA fragmentation assay of TPC-1 cell treated with escalated doses of harmine. Cells were treated with different concentrations of harmine (4, 8, and 16 *μ*g/mL) for 36 h and then total DNA was extracted and electrophoresed.

As shown in Figure 2, it is obvious to locate the characterized morphology of apoptotic cells in harmine treated group with brighter DAPI staining with condensed chromatin forming crescent-shaped profiles around the periphery of the nucleus or separate globular structures (apoptotic bodies). Quantitative analysis indicated the significant differences among the three groups treated with elevated doses of harmine, which was in accordance with the results in cytotoxicity tests (Figure 3).  Figure 4 indicated that harmine was effective to induce the fragmentation of DNA of TPC-1 cells in a dose-dependent manner, demonstrating the apoptosis inducing effect.

### 3.2. Effect of Harmine on Apoptotic Proteins and the Activity of Caspase-3

**Figure 5:**
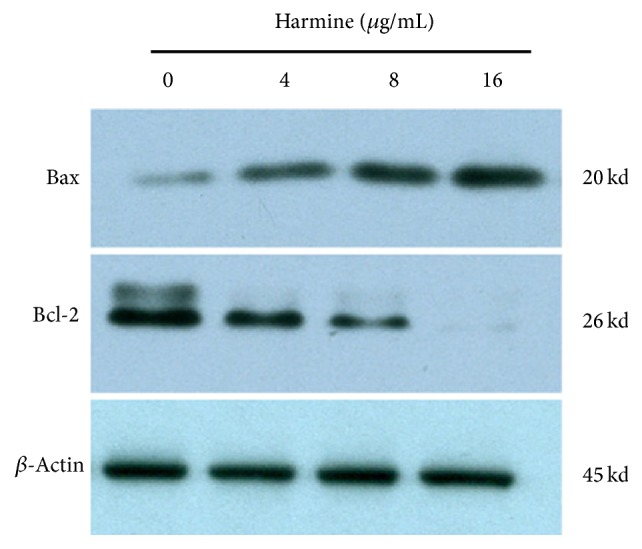
Protein expression of TPC-1 cells treated with different doses of harmine.

To examine the possible mechanism of apoptotic induction of harmine, the expression of apoptosis related proteins was evaluated by western blot. As shown in Figure 5, treatment of harmine dose-dependently induced the expression of Bax and degraded the existing Bcl-2, leading to a significant decrease in the ratio of Bcl-2/Bax, which demonstrated the progression of apoptosis.

**Figure 6:**
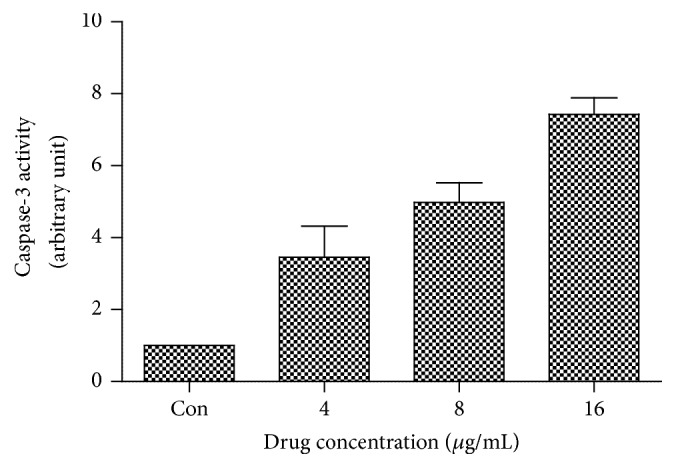
The activity of Caspase-3 in TPC-1 cells treated with different doses of harmine.

The activity of Caspase-3 in TPC-1 cells treated with a series of doses of harmine was evaluated by the caspase colorimetric protease assay kit. As one of the crucial mediators of apoptosis, Caspase-3 is considered as an apoptotic marker due to its significant role in catalyzing the specific cleavage of key proteins and generation of apoptotic bodies.  Figure 6 showed that harmine was effective in elevating the activity of Caspase-3, which was in accordance with the results of apoptosis detection from Figures 2 and 3.

### 3.3. Effect of Harmine on the Colony Forming Ability of TPC-1 Cells with or without the Combination of Radiation

**Figure 7:**
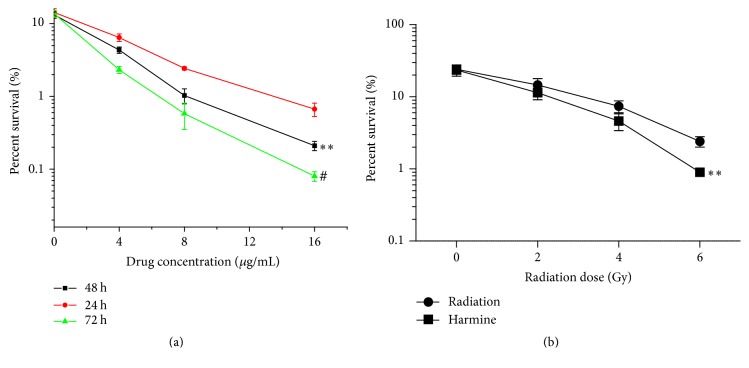
Clonogenic assay of TPC-1 cells treated with harmine and radiation. (a) Inhibitory effect of different doses of harmine on the colony forming ability of TPC-1 cells. *∗∗* represents *p* < 0.01 versus 24 h group. # represents *p* < 0.01 versus 48 h group. (b) Radiosensitization effect of harmine (2 *μ*g/mL) on TPC-1 cells. *∗∗* represents *p* < 0.01 versus radiation group.

Figure 7(a) showed the clonogenic assay of harmine on TPC-1 cells. The colony forming ability of TPC-1 cells was time- and dose-dependently inhibited by harmine. The percent survival of TPC-1 cells decreased as the concentration of harmine increased and incubation time extended, demonstrating that the antiproliferative effect of harmine was consistent with the results from cytotoxicity test.

Figure 7(b) indicated that 2 *μ*g/mL harmine effectively enhanced the inhibitory effect of radiation on the colony forming ability of TPC-1 cells. There was a significant difference between the percent survival of radiation alone and radiation plus harmine, which demonstrated the potential radiosensitizing effect of harmine.

### 3.4. Effects of Harmine on the Migration and Invasion of TPC-1 Cells

**Figure 8:**
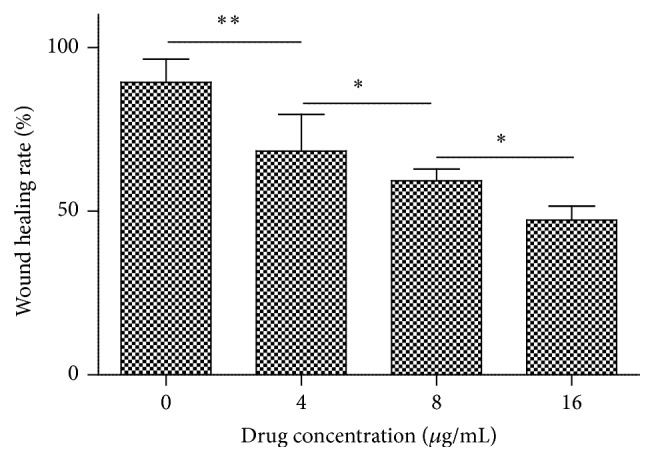
Wound healing ability of TPC-1 cells exposed to a series of doses of harmine. Quantification of cell migration using the monolayer wound scratching assay. *∗* represents *p* < 0.05. *∗* *∗* represents *p* < 0.01.

**Figure 9:**
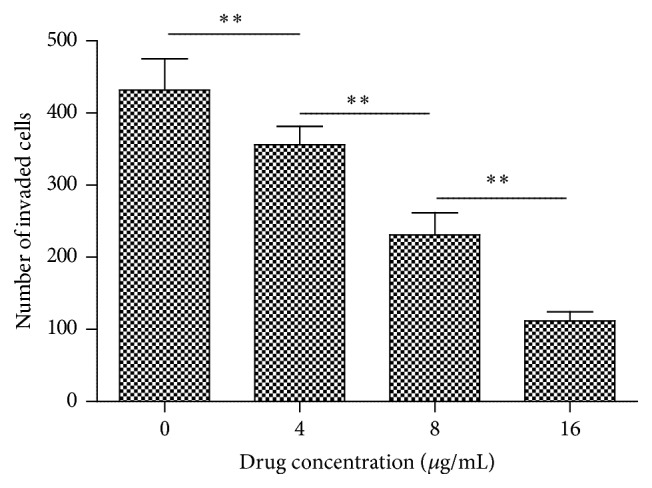
Cell invasive ability of TPC-1 cells exposed to harmine. Quantification of the numbers of invaded cells exposed to a series of doses of harmine. Each data point represents the mean ± SD from three independent experiments. *∗* *∗* represents *p* < 0.01.

Figure 8 indicated the effect of harmine on the wound healing rate of TPC-1 cells. Harmine dose-dependently decreased the wound healing rate of TPC-1 cells, which demonstrated an inhibitory effect of harmine on the migration of thyroid cancer cells. Similarly, harmine inhibited the invasion of TPC-1 cells in a dose-dependent manner as shown in Figure 9. Statistical analysis indicated significant differences among the groups treated with different doses of harmine. Similarly, the number of invading cells decreased significantly as the concentration of harmine increased. Results from wound scratch and Transwell assays demonstrate that harmine is effective in restraining the mobility of TPC-1 cells in a dose-dependent manner, which makes it a potential drug for the reverse of tumor metastasis.

### 3.5. In Vivo Antitumor Evaluation of Harmine against TPC-1 Xenograft

**Figure 10:**
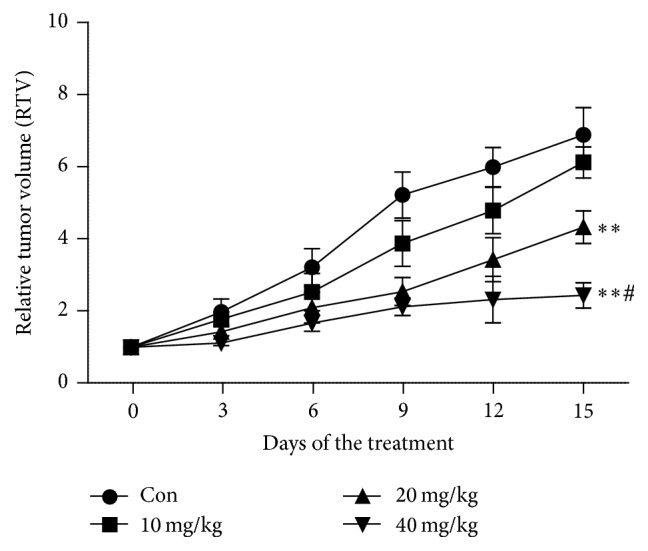
Antitumor effect of harmine in TPC-1 xenograft models. Tumor volume of established TPC-1 xenografts in nude mice during therapy under different treatments. Mice were treated with different protocols on Day 0 as shown in the figure. Saline: vehicle; harmine was administered at the doses of 10, 20, and 40 mg/kg. Different agents were delivered through intravenous pathway when tumor volume measured 100 mm^3^. Data are presented as mean ± SD (*n* = 6). The difference between tumor volumes in the group of saline and either 20 or 40 mg/kg of harmine is significant (*∗∗* means *p* < 0.01 versus control). Significant difference (# means *p* < 0.05) also is observed between the groups receiving 40 mg/kg and 20 mg/kg harmine.

In vivo antitumor effects of harmine were examined in a xenograft model of TPC-1 cells in nude mice (Figure 10). It is obvious that harmine delayed the growth of thyroid cancer in a dose-dependent manner. However, though the tumor of the mice receiving the lowest dose of harmine grew more slowly than that of the mice in the control group, there was no significant difference between the two groups. On the contrary, the other two relatively high doses of harmine significantly inhibit the growth of tumor when compared with the control group. Among all the three groups, the highest dose of harmine generated the greatest antitumor effect. Moreover, 40 mg/kg harmine restrained the tumor growth more significantly than 20 mg/kg harmine. In addition, there was no obvious toxicity (such as weight loss or behavior) in all the mice during the whole experiment.

## 4. Discussion

Here we show the cytotoxic and antiproliferative effect of harmine on the in vitro and in vivo model of thyroid cancer. As reported in previous studies, harmine showed cytotoxic and antiproliferative effect against several kinds of cancers [11, 16]. Several intracellular targets have been identified such as cyclin proteins and interference with DNA [17]. However, no report was about the antitumor effect of harmine in thyroid cancer.

In the current study, we demonstrated a time- and dose-dependent cytotoxicity of harmine against thyroid cancer cell line TPC-1. Both XTT and clonogenic assay showed that the antiproliferative effect of harmine was mainly dependent on the concentration and incubation time of harmine. Several studies have reported the dose-dependent cytotoxicity of harmine against other kinds of cancers. For example, Zhang et al. reported the time- and dose-dependent antiproliferative effect of harmine against two kinds of gastric cancer cells [11]. A recent study from South China also demonstrated the inhibitory effect of harmine derivative on the proliferation of gastric cancer cells [7].

Though most of the studies focused on the antiproliferative effect of harmine, few researchers evaluated the influence of harmine on the colony forming ability of cancer cells as well as the radiosensitizing effect. As introduced previously, loss of reproductive integrity and the inability to proliferate indefinitely is a key feature to cell death [14, 18]. Cells with integrity but unable to divide and produce a large number of colony are considered dead [19]. Therefore, cells with both the integrity and the ability to proliferate are referred to as “clonogenic,” which is pivotal to the proliferation of cancer cells and regarded as a marker of cancer malignancy [20]. Here we showed that harmine exerted a time- and dose-dependent inhibition on the clonogenic ability of TPC-1 cells, which means that harmine could effectively disrupt the reproductive integrity and lead to the inability of proliferation of thyroid cancer cells. Moreover, the current study reported for the first time that harmine showed the potential to be a promising radiosensitizer.

The following DAPI staining of cancer cells indicated that harmine was efficient to induce apoptosis of TPC-1 cells. It is known that apoptosis, also called programmed cell death, is characterized by the specific morphology, such as chromatin condensation, membrane blebbing, internucleosome degradation of DNA, and apoptotic body formation [21]. It is obvious that cells treated with harmine underwent some characteristic phenomenon of apoptosis. It is reported in earlier studies that the Bcl-2 family, including proapoptotic proteins and antiapoptotic proteins, plays an important role in apoptosis [22, 23]. Among all these proteins, the ratio of Bcl-2 and Bax is a key indicator of apoptosis [24].  Figure 4 showed that the treatment by harmine dose-dependently decreased the expression of Bcl-2 while simultaneously increased the expression of Bax, which led to an obvious decline in the ratio of Bcl-2 and Bax. Though the effect of apoptotic induction by harmine was also reported by other groups, different mechanisms underlying the antitumor effect of harmine have been identified such as cell cycle arrest and telomerase activity inhibition [9]. The anticancer effect of harmine was confirmed in TPC-1 xenograft model, which, together with other studies focusing on harmine, proved the efficacy of harmine in the treatment of cancer.

The ability of migration and invasion is the characteristic of malignancy, which often leads to the failure of cancer therapy [25]. The efficient inhibition of tumor migration and invasion is an effective way to control the metastasis of tumor. Since few studies examined the antimetastatic effect of harmine, we evaluate the ability of harmine on cell migration and invasion through wound scratching and Transwell assays. Results from the two experiments demonstrated the dose-dependent inhibitory effect of harmine in vitro. Moreover, animal experiment on metastatic xenograft models in the author's lab is ongoing to further evaluate the in vivo antimetastasis efficacy of harmine.

As mentioned above, evidences from our report not only demonstrate the potential antitumor effect of harmine, but also provide a feasible way to counteract the metastasis of thyroid cancer. Moreover, further studies focusing on the chemosensitization effect of harmine are ongoing in the author's lab to explore more potential application of such alkaloids. It is undoubtedly, however, that the development of herbal medicine, especially alkaloids, warrants more intensive research in order to evaluate the feasibility and advantages of clinical applications.

In summary, the current study demonstrates the antitumor effect of harmine in the treatment of thyroid cancer. In vitro cytotoxicity tests indicate that harmine possesses a dose- and time-dependent cell inhibitory and apoptosis inducing effect on TPC-1 cells. In addition, harmine effectively inhibits the migration and invasion of TPC-1 cells in a dose-dependent manner. Moreover, in vivo evaluation shows that harmine significantly delays the growth of thyroid cancer in a dose-dependent manner in nude mice. Therefore, harmine might be a promising herbal medicine in tumor therapy and further efforts are needed to explore this therapeutic strategy.
